# *rac*-Diethyl 5-oxo-2-[(2,4,4-trimethyl­pentan-2-yl)amino]-4,5-dihydro­pyrano[3,2-*c*]chromene-3,4-dicarboxyl­ate

**DOI:** 10.1107/S1600536811051786

**Published:** 2011-12-14

**Authors:** S. Antony Inglebert, K. Sethusankar, Yuvaraj Arun, Paramasivam T. Perumal

**Affiliations:** a Department of Physics, Sri Ram Engineering College, Chennai 602 024, India; b Department of Physics, RKM Vivekananda College (Autonomous), Chennai 600 004, India; c Organic Chemistry Division, Central Leather Research Institute, Adyar, Chennai 600 020, India

## Abstract

The title compound, C_26_H_33_NO_7_, comprises a racemic mixture of asymmetric mol­ecules containing one stereogenic centre. The dihedral angle between the mean planes of the fused pyran ring and the coumarin ring system is 8.12 (14)°. The mol­ecular structure features a short N—H⋯O contact, which generates an *S*(6) ring motif. The crystal packing are stabilized by C—H⋯O inter­actions.

## Related literature

For a related structure, see: Inglebert *et al.* (2011 ▶). For general background and applications of coumarin derivatives, see: Griffiths *et al.* (1995 ▶); Yu *et al.* (2006 ▶). For graph-set notation, see: Bernstein *et al.* (1995 ▶).
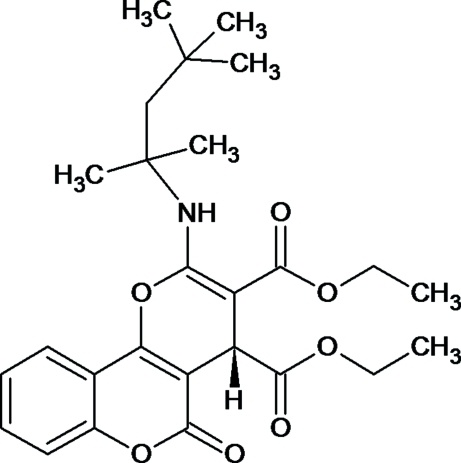


## Experimental

### 

#### Crystal data

C_26_H_33_NO_7_
*M* *_r_* = 471.53
Orthorhombic, 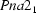

*a* = 11.6910 (17) Å
*b* = 18.786 (3) Å
*c* = 11.7305 (15) Å
*V* = 2576.3 (6) Å^3^
*Z* = 4
Mo *K*α radiation
μ = 0.09 mm^−1^
*T* = 295 K
0.30 × 0.25 × 0.20 mm

#### Data collection

Bruker Kappa APEXII CCD diffractometer
Absorption correction: multi-scan (*SADABS*; Bruker, 2008 ▶) *T*_min_ = 0.974, *T*_max_ = 0.983
10574 measured reflections
3699 independent reflections
2668 reflections with *I* > 2σ(*I*)
*R*_int_ = 0.040

#### Refinement

*R*[*F* ^2^ > 2σ(*F* ^2^)] = 0.049
*wR*(*F* ^2^) = 0.148
*S* = 1.09
3699 reflections
314 parameters
1 restraint
H-atom parameters constrained
Δρ_max_ = 0.31 e Å^−3^
Δρ_min_ = −0.25 e Å^−3^

### 

Data collection: *APEX2* (Bruker, 2008 ▶); cell refinement: *SAINT* (Bruker, 2008 ▶); data reduction: *SAINT*; program(s) used to solve structure: *SHELXS97* (Sheldrick, 2008 ▶); program(s) used to refine structure: *SHELXL97* (Sheldrick, 2008 ▶); molecular graphics: *ORTEP-3* (Farrugia, 1997 ▶); software used to prepare material for publication: *SHELXL97* and *PLATON* (Spek, 2009 ▶).

## Supplementary Material

Crystal structure: contains datablock(s) global, I. DOI: 10.1107/S1600536811051786/rk2310sup1.cif

Structure factors: contains datablock(s) I. DOI: 10.1107/S1600536811051786/rk2310Isup2.hkl

Supplementary material file. DOI: 10.1107/S1600536811051786/rk2310Isup3.cml

Additional supplementary materials:  crystallographic information; 3D view; checkCIF report

## Figures and Tables

**Table 1:** Hydrogen-bond geometry (Å, °)

*D*—H⋯*A*	*D*—H	H⋯*A*	*D*⋯*A*	*D*—H⋯*A*
N1—H1⋯O6	0.86	1.99	2.659 (4)	135
C2—H2⋯O6^i^	0.93	2.47	3.252 (5)	141
C4—H4⋯O4^ii^	0.93	2.43	3.306 (5)	157

Symmetry codes: (i) 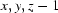; (ii) 
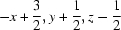
.

**Fig. 1.**
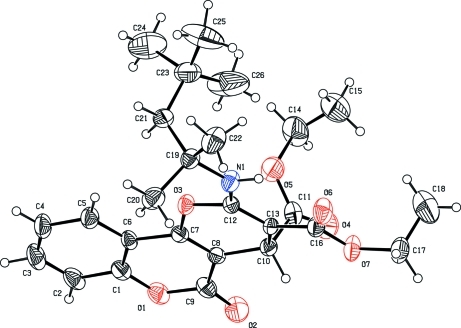
The part of molecular structure of the title compound, showing the atom numbering scheme. Displacement ellipsoids are drawn at 30% probability level. H atoms are present as small spheres of arbitary radius.

**Fig. 2.**
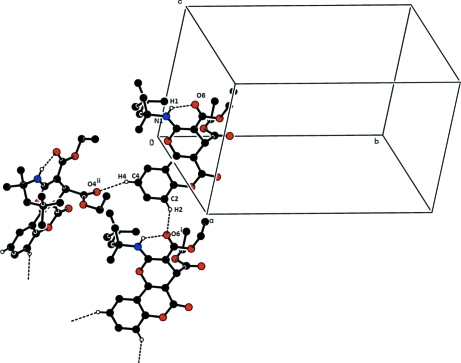
The packing structure of the title compound viewed along the a axis. Figure shows that intermolecular C–H···O and intramolecular N–H···O interactions as dashed lines. H atoms have omited for clarity.

## supplementary crystallographic information

### Comment

Coumarin and its derivatives have been extensively used in industrial products as dyes/laser materials, photosensitizers, pestisides, in pharmacology and in enzymology as biological probes. The photophysical and spectroscopic properties of the coumarin derivatives can be readily modified by the introduction of substituents in parent coumarin, converting themselves into more useful products and more flexibility to fit well in various applications (Griffiths *et al.*, 1995; Yu *et al.*, 2006).

In the title compound, C_26_H_33_NO_7_, the coumarin ring system is attached to a pyran ring, two diethyl carboxylates and the mean plane of tri methyl pentan amine group. The coumarin ring system is almost planar with a maximum deviation of -0.011 (4)Å for C8 atom and minimal puckering. Total puckering amplitude of coumarin ring system is 0.016 (4)Å. The coumarin ring system (O1/C1-C9) makes a dihedral angle of 8.12 (14)° with the pyran ring (O3/C7/C8/C10-C12). The coumarin ring system forms dihedral angles of 55.83 (15)° and 16.80 (9)° with the ethyl carboxylates (C13/O4/O5/C14/C15) and (C10/O6/O7/C17/C18) respectively. Likewise the pyran ring forms dihedral angles of 65.08 (18)° and 9.83 (11)° with the ethyl carboxylates (C13/O4/O5/C14/C15) and (C10/O6/O7/C17/C18) respectively. The dihedral angle between two ethyl carboxylate group is 72.93 (15)°.

The molecule is chiral with an asymmetric center (atom C10) present in the pyran ring. The nitrogen atom N1 deviates by -0.1146 (31)° from the pyran ring and carbon atom the C21 deviates by 1.8344 (37)° and 1.0411 (36)° from the mean plane of the butyl and pyran ring. The title compound exhibits structural similarities with a previously reported structure (Inglebert *et al.*, 2011).

The molecular structure features a short intramolecular N1–H1···O6 contact, which generates an *S*(6) ring motif (Bernstein *et al.*, 1995). The hydrogen bond is bifurcated, with oxygen O5 being simultaneously donated to two equivalent H atoms, forming one intramolecular (N1–H1···O6) and one intermolecular (C2–H2···O6^i^) hydrogen bonds. The crystal packing is further stabilized by C4–H4···O4^ii^ intermolecular hydrogen bonds (Table 1). The symmetry codes: (i) *x*, *y*, -1+*z*; (ii) 3/2-*x*, 1/2+*y*, -1/2+ *z*.

### Experimental

To a magnetically stirred solution of 4-hydroxy coumarin (0.162 g, 1.0 mmol) and diethyl acetylenedicarboxylate (0.170 g, 1.0 mmol) in CH_3_CN (10 ml) was added a solution of 1,1,3,3-tetra methylbutyl isocynaide (0.139 g, 1.0 mmol) at room temperature over 5 min. The mixture was then stirred for 24 h. After completion of the reaction, the solvent was removed under vacuum and the solid residue was washed with *n*-hexane and crystallized from CH_2_Cl_2_/*n*-hexane(1/2) to give product as white crystals (0.396 g, 84%).

### Refinement

Positions of hydrogen atoms were localized from the difference electron density maps and their distances were geometrically constrained. The H atoms bound to the C and N atoms were treated as riding atoms, with N–H = 0.86Å and *U*_iso_(H) = 1.2*U*_eq_(N) for amine group, with C–H = 0.93Å and *U*_iso_(H) = 1.2*U*_eq_(C) for aromatic, C–H = 0.97Å and *U*_iso_(H) = 1.2*U*_eq_(C) for methylene and C–H = 0.96Å and *U*_iso_(H) = 1.5*U*_eq_(C) for methyl groups. The rotation angles for methyl groups were optimized by least squares.

In the diffraction experiment were measured 1388 Fridedel pairs. Because no heavy atoms (*Z* > Si) in the molecule, during the refinement by *SHELXL97*, was used 'MERG 2' instruction in final CIF descriptors were placed: _refine_ls_abs_structure_Flack "?".

### Crystal data

**Table:**

C_26_H_33_NO_7_	*F*(000) = 1008
*M**_r_* = 471.53	*D*_x_ = 1.216 Mg m^−^^3^
Orthorhombic, *P**n**a*2_1_	Mo *K*α radiation, λ = 0.71073 Å
Hall symbol: P 2c -2n	Cell parameters from 3699 reflections
*a* = 11.6910 (17) Å	θ = 2.1–24.7°
*b* = 18.786 (3) Å	µ = 0.09 mm^−^^1^
*c* = 11.7305 (15) Å	*T* = 295 K
*V* = 2576.3 (6) Å^3^	Block, colourless
*Z* = 4	0.30 × 0.25 × 0.20 mm

### Data collection

**Table:**

Bruker Kappa APEXII CCD diffractometer	3699 independent reflections
Radiation source: fine-focus sealed tube	2668 reflections with *I* > 2σ(*I*)
graphite	*R*_int_ = 0.040
ω and φ scans	θ_max_ = 24.7°, θ_min_ = 2.1°
Absorption correction: multi-scan (*SADABS*; Bruker, 2008)	*h* = −13→12
*T*_min_ = 0.974, *T*_max_ = 0.983	*k* = −22→21
10574 measured reflections	*l* = −13→8

### Refinement

**Table:**

Refinement on *F*^2^	Primary atom site location: structure-invariant direct methods
Least-squares matrix: full	Secondary atom site location: difference Fourier map
*R*[*F*^2^ > 2σ(*F*^2^)] = 0.049	Hydrogen site location: inferred from neighbouring sites
*wR*(*F*^2^) = 0.148	H-atom parameters constrained
*S* = 1.09	*w* = 1/[σ^2^(*F*_o_^2^) + (0.078*P*)^2^ + 0.3187*P*] where *P* = (*F*_o_^2^ + 2*F*_c_^2^)/3
3699 reflections	(Δ/σ)_max_ < 0.001
314 parameters	Δρ_max_ = 0.31 e Å^−^^3^
1 restraint	Δρ_min_ = −0.25 e Å^−^^3^

### Special details

**Table:**

Geometry. All s.u.'s (except the s.u.'s in the dihedral angle between two l.s. planes) are estimated using the full covariance matrix. The cell s.u.'s are taken into account individually in the estimation of s.u.'s in distances, angles and torsion angles; correlations between s.u.'s in cell parameters are only used when they are defined by crystal symmetry. An approximate (isotropic) treatment of cell s.u.'s is used for estimating s.u.'s involving l.s. planes.
Refinement. Refinement of *F*^2^ against ALL reflections. The weighted *R*-factor *wR* and goodness of fit *S* are based on *F*^2^, conventional *R*-factors *R* are based on *F*, with *F* set to zero for negative *F*^2^. The threshold expression of *F*^2^ > σ(*F*^2^) is used only for calculating *R*-factors(gt) *etc*. and is not relevant to the choice of reflections for refinement. *R*-factors based on *F*^2^ are statistically about twice as large as those based on *F*, and *R*-factors based on ALL data will be even larger.

### Fractional atomic coordinates and isotropic or equivalent isotropic displacement parameters (Å^2^)

**Table:**

	*x*	*y*	*z*	*U*_iso_*/*U*_eq_	
C1	0.6377 (3)	−0.0116 (2)	0.3451 (3)	0.0508 (10)	
C2	0.6418 (4)	0.0147 (3)	0.2347 (4)	0.0644 (12)	
H2	0.6107	−0.0109	0.1743	0.077*	
C3	0.6927 (4)	0.0791 (3)	0.2170 (4)	0.0755 (14)	
H3	0.6946	0.0975	0.1435	0.091*	
C4	0.7414 (4)	0.1179 (3)	0.3041 (4)	0.0683 (13)	
H4	0.7766	0.1614	0.2894	0.082*	
C5	0.7370 (3)	0.0910 (2)	0.4138 (3)	0.0541 (10)	
H5	0.7691	0.1167	0.4736	0.065*	
C6	0.6849 (3)	0.02601 (18)	0.4351 (3)	0.0423 (9)	
C7	0.6755 (3)	−0.00743 (18)	0.5456 (3)	0.0394 (8)	
C8	0.6263 (3)	−0.07045 (18)	0.5616 (3)	0.0395 (8)	
C9	0.5766 (3)	−0.1081 (2)	0.4650 (4)	0.0538 (10)	
C10	0.6226 (3)	−0.10563 (18)	0.6758 (3)	0.0427 (8)	
H10	0.5457	−0.1252	0.6872	0.051*	
C11	0.6445 (3)	−0.05068 (18)	0.7671 (3)	0.0385 (8)	
C12	0.6961 (3)	0.01298 (17)	0.7433 (3)	0.0402 (8)	
C13	0.7083 (4)	−0.1669 (2)	0.6807 (4)	0.0573 (10)	
C14	0.9084 (5)	−0.1933 (3)	0.6863 (6)	0.108 (2)	
H14A	0.9755	−0.1771	0.6450	0.129*	
H14B	0.8860	−0.2391	0.6553	0.129*	
C15	0.9368 (6)	−0.2013 (4)	0.8074 (7)	0.123 (2)	
H15A	0.9581	−0.1559	0.8383	0.184*	
H15B	0.9994	−0.2340	0.8155	0.184*	
H15C	0.8714	−0.2192	0.8478	0.184*	
C16	0.6163 (3)	−0.06638 (19)	0.8839 (3)	0.0493 (9)	
C17	0.5375 (5)	−0.1526 (3)	1.0102 (4)	0.0780 (14)	
H17A	0.5140	−0.1114	1.0541	0.094*	
H17B	0.4738	−0.1857	1.0074	0.094*	
C18	0.6319 (7)	−0.1854 (4)	1.0635 (7)	0.147 (3)	
H18A	0.6500	−0.2289	1.0245	0.221*	
H18B	0.6133	−0.1957	1.1415	0.221*	
H18C	0.6966	−0.1540	1.0608	0.221*	
C19	0.7794 (3)	0.13287 (19)	0.8039 (3)	0.0497 (9)	
C20	0.6949 (3)	0.1798 (2)	0.7392 (5)	0.0683 (12)	
H20A	0.6810	0.1598	0.6652	0.102*	
H20B	0.7262	0.2268	0.7310	0.102*	
H20C	0.6242	0.1824	0.7807	0.102*	
C21	0.8934 (3)	0.13309 (19)	0.7391 (4)	0.0559 (10)	
H21A	0.9180	0.1824	0.7360	0.067*	
H21B	0.8756	0.1195	0.6614	0.067*	
C22	0.7911 (5)	0.1623 (2)	0.9242 (4)	0.0801 (15)	
H22A	0.7166	0.1721	0.9547	0.120*	
H22B	0.8352	0.2054	0.9224	0.120*	
H22C	0.8290	0.1279	0.9715	0.120*	
C23	1.0000 (4)	0.0895 (3)	0.7732 (5)	0.0871 (16)	
C24	1.0869 (5)	0.1021 (5)	0.6791 (9)	0.166 (3)	
H24A	1.1541	0.0742	0.6936	0.249*	
H24B	1.1070	0.1517	0.6770	0.249*	
H24C	1.0545	0.0885	0.6071	0.249*	
C25	1.0573 (6)	0.1221 (5)	0.8796 (8)	0.177 (4)	
H25A	1.1344	0.1050	0.8856	0.266*	
H25B	1.0152	0.1086	0.9465	0.266*	
H25C	1.0579	0.1731	0.8730	0.266*	
C26	0.9782 (6)	0.0111 (3)	0.7841 (11)	0.197 (5)	
H26A	0.9416	−0.0060	0.7161	0.295*	
H26B	0.9297	0.0024	0.8486	0.295*	
H26C	1.0496	−0.0133	0.7944	0.295*	
O1	0.5851 (3)	−0.07650 (15)	0.3600 (2)	0.0612 (7)	
O2	0.5283 (3)	−0.16455 (17)	0.4702 (3)	0.0809 (9)	
O3	0.7213 (2)	0.03244 (13)	0.6328 (2)	0.0469 (6)	
O4	0.6857 (3)	−0.22770 (16)	0.6899 (4)	0.0995 (12)	
O5	0.8155 (3)	−0.14240 (16)	0.6705 (3)	0.0857 (11)	
O6	0.6299 (3)	−0.02675 (14)	0.9658 (2)	0.0674 (8)	
O7	0.5678 (3)	−0.13089 (14)	0.8956 (2)	0.0648 (8)	
N1	0.7291 (3)	0.06126 (16)	0.8183 (3)	0.0531 (8)	
H1	0.7195	0.0488	0.8881	0.064*	

### Atomic displacement parameters (Å^2^)

**Table:**

	*U*^11^	*U*^22^	*U*^33^	*U*^12^	*U*^13^	*U*^23^
C1	0.047 (2)	0.065 (3)	0.041 (2)	0.0145 (19)	0.0032 (19)	0.0016 (19)
C2	0.061 (3)	0.097 (3)	0.035 (2)	0.022 (2)	0.000 (2)	0.005 (2)
C3	0.080 (3)	0.104 (4)	0.043 (3)	0.029 (3)	0.017 (2)	0.026 (3)
C4	0.069 (3)	0.079 (3)	0.057 (3)	0.013 (2)	0.019 (2)	0.029 (3)
C5	0.057 (2)	0.060 (2)	0.046 (3)	0.004 (2)	0.0088 (19)	0.0084 (19)
C6	0.0436 (19)	0.050 (2)	0.034 (2)	0.0126 (17)	0.0063 (16)	0.0075 (17)
C7	0.0359 (19)	0.0484 (19)	0.034 (2)	0.0050 (16)	0.0016 (16)	−0.0002 (17)
C8	0.0358 (19)	0.0450 (19)	0.038 (2)	0.0029 (16)	−0.0019 (16)	0.0002 (15)
C9	0.062 (3)	0.055 (2)	0.045 (2)	0.004 (2)	−0.012 (2)	−0.003 (2)
C10	0.044 (2)	0.045 (2)	0.038 (2)	−0.0045 (16)	−0.0007 (17)	0.0013 (15)
C11	0.0355 (18)	0.0477 (19)	0.032 (2)	−0.0039 (16)	0.0013 (15)	0.0071 (15)
C12	0.0406 (19)	0.0470 (19)	0.033 (2)	−0.0018 (16)	−0.0018 (18)	0.0036 (17)
C13	0.076 (3)	0.046 (2)	0.050 (2)	0.003 (2)	−0.001 (2)	0.0082 (18)
C14	0.097 (4)	0.095 (4)	0.131 (5)	0.052 (3)	0.024 (4)	0.031 (4)
C15	0.109 (5)	0.119 (5)	0.140 (6)	0.028 (4)	−0.011 (4)	0.025 (4)
C16	0.055 (2)	0.051 (2)	0.042 (2)	−0.0062 (18)	0.0017 (19)	0.0048 (18)
C17	0.092 (4)	0.078 (3)	0.063 (3)	−0.022 (3)	0.001 (3)	0.022 (2)
C18	0.143 (6)	0.158 (7)	0.140 (7)	0.025 (5)	−0.016 (5)	0.066 (6)
C19	0.053 (2)	0.047 (2)	0.049 (2)	−0.0072 (18)	0.0023 (19)	−0.0012 (17)
C20	0.059 (3)	0.058 (2)	0.088 (3)	0.001 (2)	0.007 (3)	0.004 (2)
C21	0.051 (2)	0.053 (2)	0.064 (3)	−0.0103 (17)	−0.010 (2)	0.0040 (19)
C22	0.100 (4)	0.075 (3)	0.065 (3)	−0.033 (3)	0.003 (3)	−0.015 (2)
C23	0.056 (3)	0.080 (3)	0.126 (5)	−0.003 (2)	−0.016 (3)	0.011 (3)
C24	0.068 (4)	0.217 (9)	0.214 (9)	0.036 (5)	0.029 (5)	0.018 (7)
C25	0.116 (6)	0.229 (9)	0.188 (9)	0.017 (6)	−0.090 (6)	−0.013 (8)
C26	0.086 (4)	0.079 (4)	0.425 (17)	0.010 (3)	−0.060 (7)	0.042 (7)
O1	0.0684 (18)	0.0741 (19)	0.0410 (17)	−0.0001 (15)	−0.0120 (14)	−0.0039 (14)
O2	0.103 (2)	0.0674 (18)	0.072 (2)	−0.0260 (18)	−0.026 (2)	−0.0017 (17)
O3	0.0557 (15)	0.0522 (14)	0.0328 (14)	−0.0126 (12)	0.0012 (12)	0.0036 (12)
O4	0.109 (3)	0.0437 (17)	0.145 (3)	0.0013 (18)	−0.018 (2)	0.0142 (18)
O5	0.065 (2)	0.0698 (18)	0.122 (3)	0.0217 (17)	0.0166 (19)	0.0305 (18)
O6	0.096 (2)	0.0688 (17)	0.0373 (17)	−0.0171 (16)	0.0010 (16)	0.0005 (14)
O7	0.0885 (19)	0.0589 (16)	0.0470 (17)	−0.0158 (15)	0.0058 (15)	0.0127 (13)
N1	0.067 (2)	0.0570 (19)	0.0351 (17)	−0.0181 (16)	−0.0014 (16)	−0.0014 (15)

### Geometric parameters (Å, °)

**Table:**

C1—O1	1.377 (5)	C16—O7	1.345 (4)
C1—C6	1.385 (5)	C17—C18	1.409 (8)
C1—C2	1.386 (6)	C17—O7	1.449 (5)
C2—C3	1.364 (6)	C17—H17A	0.9700
C2—H2	0.9300	C17—H17B	0.9700
C3—C4	1.378 (7)	C18—H18A	0.9600
C3—H3	0.9300	C18—H18B	0.9600
C4—C5	1.383 (6)	C18—H18C	0.9600
C4—H4	0.9300	C19—N1	1.478 (5)
C5—C6	1.388 (5)	C19—C22	1.522 (6)
C5—H5	0.9300	C19—C20	1.526 (6)
C6—C7	1.445 (5)	C19—C21	1.535 (6)
C7—C8	1.329 (4)	C20—H20A	0.9600
C7—O3	1.376 (4)	C20—H20B	0.9600
C8—C9	1.457 (5)	C20—H20C	0.9600
C8—C10	1.494 (5)	C21—C23	1.543 (6)
C9—O2	1.203 (4)	C21—H21A	0.9700
C9—O1	1.372 (5)	C21—H21B	0.9700
C10—C11	1.509 (5)	C22—H22A	0.9600
C10—C13	1.528 (5)	C22—H22B	0.9600
C10—H10	0.9800	C22—H22C	0.9600
C11—C12	1.368 (5)	C23—C26	1.500 (8)
C11—C16	1.440 (5)	C23—C24	1.520 (10)
C12—N1	1.321 (5)	C23—C25	1.543 (10)
C12—O3	1.378 (4)	C24—H24A	0.9600
C13—O4	1.177 (4)	C24—H24B	0.9600
C13—O5	1.340 (5)	C24—H24C	0.9600
C14—O5	1.459 (5)	C25—H25A	0.9600
C14—C15	1.467 (9)	C25—H25B	0.9600
C14—H14A	0.9700	C25—H25C	0.9600
C14—H14B	0.9700	C26—H26A	0.9600
C15—H15A	0.9600	C26—H26B	0.9600
C15—H15B	0.9600	C26—H26C	0.9600
C15—H15C	0.9600	N1—H1	0.8600
C16—O6	1.226 (5)		
			
O1—C1—C6	122.2 (3)	H17A—C17—H17B	108.2
O1—C1—C2	116.7 (4)	C17—C18—H18A	109.5
C6—C1—C2	121.1 (4)	C17—C18—H18B	109.5
C3—C2—C1	118.2 (4)	H18A—C18—H18B	109.5
C3—C2—H2	120.9	C17—C18—H18C	109.5
C1—C2—H2	120.9	H18A—C18—H18C	109.5
C2—C3—C4	122.5 (4)	H18B—C18—H18C	109.5
C2—C3—H3	118.8	N1—C19—C22	105.1 (3)
C4—C3—H3	118.8	N1—C19—C20	109.0 (3)
C3—C4—C5	118.7 (4)	C22—C19—C20	108.1 (4)
C3—C4—H4	120.6	N1—C19—C21	113.9 (3)
C5—C4—H4	120.6	C22—C19—C21	112.3 (4)
C4—C5—C6	120.4 (4)	C20—C19—C21	108.3 (3)
C4—C5—H5	119.8	C19—C20—H20A	109.5
C6—C5—H5	119.8	C19—C20—H20B	109.5
C1—C6—C5	119.1 (4)	H20A—C20—H20B	109.5
C1—C6—C7	115.6 (3)	C19—C20—H20C	109.5
C5—C6—C7	125.3 (3)	H20A—C20—H20C	109.5
C8—C7—O3	123.2 (3)	H20B—C20—H20C	109.5
C8—C7—C6	123.1 (3)	C19—C21—C23	124.8 (4)
O3—C7—C6	113.6 (3)	C19—C21—H21A	106.1
C7—C8—C9	119.7 (3)	C23—C21—H21A	106.1
C7—C8—C10	122.2 (3)	C19—C21—H21B	106.1
C9—C8—C10	118.1 (3)	C23—C21—H21B	106.1
O2—C9—O1	117.4 (4)	H21A—C21—H21B	106.3
O2—C9—C8	125.2 (4)	C19—C22—H22A	109.5
O1—C9—C8	117.4 (3)	C19—C22—H22B	109.5
C8—C10—C11	109.2 (3)	H22A—C22—H22B	109.5
C8—C10—C13	110.3 (3)	C19—C22—H22C	109.5
C11—C10—C13	112.2 (3)	H22A—C22—H22C	109.5
C8—C10—H10	108.3	H22B—C22—H22C	109.5
C11—C10—H10	108.3	C26—C23—C24	109.2 (7)
C13—C10—H10	108.3	C26—C23—C21	114.0 (4)
C12—C11—C16	118.3 (3)	C24—C23—C21	105.6 (5)
C12—C11—C10	121.9 (3)	C26—C23—C25	113.2 (7)
C16—C11—C10	119.8 (3)	C24—C23—C25	103.6 (6)
N1—C12—C11	126.4 (3)	C21—C23—C25	110.5 (5)
N1—C12—O3	112.4 (3)	C23—C24—H24A	109.5
C11—C12—O3	121.2 (3)	C23—C24—H24B	109.5
O4—C13—O5	123.5 (4)	H24A—C24—H24B	109.5
O4—C13—C10	126.0 (4)	C23—C24—H24C	109.5
O5—C13—C10	110.5 (3)	H24A—C24—H24C	109.5
O5—C14—C15	111.0 (5)	H24B—C24—H24C	109.5
O5—C14—H14A	109.4	C23—C25—H25A	109.5
C15—C14—H14A	109.4	C23—C25—H25B	109.5
O5—C14—H14B	109.4	H25A—C25—H25B	109.5
C15—C14—H14B	109.4	C23—C25—H25C	109.5
H14A—C14—H14B	108.0	H25A—C25—H25C	109.5
C14—C15—H15A	109.5	H25B—C25—H25C	109.5
C14—C15—H15B	109.5	C23—C26—H26A	109.5
H15A—C15—H15B	109.5	C23—C26—H26B	109.5
C14—C15—H15C	109.5	H26A—C26—H26B	109.5
H15A—C15—H15C	109.5	C23—C26—H26C	109.5
H15B—C15—H15C	109.5	H26A—C26—H26C	109.5
O6—C16—O7	121.4 (3)	H26B—C26—H26C	109.5
O6—C16—C11	126.3 (3)	C9—O1—C1	122.0 (3)
O7—C16—C11	112.2 (3)	C7—O3—C12	118.1 (2)
C18—C17—O7	110.1 (5)	C13—O5—C14	117.3 (4)
C18—C17—H17A	109.6	C16—O7—C17	116.9 (3)
O7—C17—H17A	109.6	C12—N1—C19	131.7 (3)
C18—C17—H17B	109.6	C12—N1—H1	114.2
O7—C17—H17B	109.6	C19—N1—H1	114.2
			
O1—C1—C2—C3	179.6 (3)	C10—C11—C12—O3	5.9 (5)
C6—C1—C2—C3	−0.6 (6)	C8—C10—C13—O4	−114.4 (5)
C1—C2—C3—C4	1.1 (6)	C11—C10—C13—O4	123.6 (5)
C2—C3—C4—C5	−1.0 (6)	C8—C10—C13—O5	64.3 (4)
C3—C4—C5—C6	0.3 (6)	C11—C10—C13—O5	−57.7 (4)
O1—C1—C6—C5	179.8 (3)	C12—C11—C16—O6	3.1 (6)
C2—C1—C6—C5	0.0 (5)	C10—C11—C16—O6	179.8 (4)
O1—C1—C6—C7	0.1 (5)	C12—C11—C16—O7	−179.0 (3)
C2—C1—C6—C7	−179.7 (3)	C10—C11—C16—O7	−2.3 (5)
C4—C5—C6—C1	0.1 (5)	N1—C19—C21—C23	−55.7 (5)
C4—C5—C6—C7	179.8 (3)	C22—C19—C21—C23	63.6 (5)
C1—C6—C7—C8	0.8 (5)	C20—C19—C21—C23	−177.1 (4)
C5—C6—C7—C8	−178.9 (3)	C19—C21—C23—C26	55.6 (8)
C1—C6—C7—O3	−178.7 (3)	C19—C21—C23—C24	175.4 (5)
C5—C6—C7—O3	1.7 (5)	C19—C21—C23—C25	−73.2 (6)
O3—C7—C8—C9	178.0 (3)	O2—C9—O1—C1	179.3 (3)
C6—C7—C8—C9	−1.4 (5)	C8—C9—O1—C1	−0.3 (5)
O3—C7—C8—C10	−3.7 (5)	C6—C1—O1—C9	−0.3 (5)
C6—C7—C8—C10	176.9 (3)	C2—C1—O1—C9	179.5 (4)
C7—C8—C9—O2	−178.4 (4)	C8—C7—O3—C12	−12.7 (4)
C10—C8—C9—O2	3.2 (6)	C6—C7—O3—C12	166.8 (3)
C7—C8—C9—O1	1.1 (5)	N1—C12—O3—C7	−169.8 (3)
C10—C8—C9—O1	−177.3 (3)	C11—C12—O3—C7	11.3 (4)
C7—C8—C10—C11	18.6 (4)	O4—C13—O5—C14	−7.9 (7)
C9—C8—C10—C11	−163.1 (3)	C10—C13—O5—C14	173.3 (4)
C7—C8—C10—C13	−105.2 (4)	C15—C14—O5—C13	−84.9 (6)
C9—C8—C10—C13	73.2 (4)	O6—C16—O7—C17	−2.9 (5)
C8—C10—C11—C12	−19.6 (4)	C11—C16—O7—C17	179.0 (4)
C13—C10—C11—C12	103.0 (4)	C18—C17—O7—C16	−86.8 (6)
C8—C10—C11—C16	163.8 (3)	C11—C12—N1—C19	−176.2 (3)
C13—C10—C11—C16	−73.6 (4)	O3—C12—N1—C19	4.9 (5)
C16—C11—C12—N1	3.8 (5)	C22—C19—N1—C12	176.9 (4)
C10—C11—C12—N1	−172.9 (3)	C20—C19—N1—C12	61.3 (5)
C16—C11—C12—O3	−177.4 (3)	C21—C19—N1—C12	−59.8 (5)

### Hydrogen-bond geometry (Å, °)

**Table:**

*D*—H···*A*	*D*—H	H···*A*	*D*···*A*	*D*—H···*A*
N1—H1···O6	0.86	1.99	2.659 (4)	135
C2—H2···O6^i^	0.93	2.47	3.252 (5)	141.
C4—H4···O4^ii^	0.93	2.43	3.306 (5)	157.

Symmetry codes: (i) *x*, *y*, *z*−1; (ii) −*x*+3/2, *y*+1/2, *z*−1/2.
